# The impact of introducing tyrosine kinase inhibitors on chronic myeloid leukemia survival: a population-based study

**DOI:** 10.1186/s12885-018-4984-3

**Published:** 2018-11-06

**Authors:** Enza Di Felice, Francesca Roncaglia, Francesco Venturelli, Lucia Mangone, Stefano Luminari, Claudia Cirilli, Giuliano Carrozzi, Paolo Giorgi Rossi

**Affiliations:** 1 Epidemiology Unit, Azienda USL-IRCCS di Reggio Emilia, Reggio Emilia, Italy; 2 Department of Health, Emilia-Romagna Region, Bologna, Italy; 3 Clinical and Experimental Medicine PhD Program, University of Modena and Reggio Emilia, Modena, Italy; 4 Hematology Unit, Azienda USL-IRCCS di Reggio Emilia, Reggio Emilia, Italy; 5 Department of Diagnostics, Clinical and Public Health Medicine, University of Modena and Reggio Emilia, Modena, Italy; 6 Epidemiology Unit, AUSL of Modena, Modena, Italy

**Keywords:** Chronic myeloid leukemia, Imatinib Mesylate, Survival analysis, Population registers, Interrupted time series analysis

## Abstract

**Background:**

Chronic myeloid leukemia is associated with a BCR/ABL oncoprotein inhibited by imatinib mesylate, the first tyrosine kinase inhibitor. Although experimental studies have clearly demonstrated the efficacy of imatinib, up-to-date data on its effectiveness at the population level are limited.

Our study aims to assess the change in disease-specific survival for chronic myeloid leukemia after introducing tyrosine kinase inhibitors in first-line treatment.

**Methods:**

This study analyzed data from two population-based cancer registries in Italy. Disease-specific survival for chronic myeloid leukemia cases diagnosed before and after the introduction of tyrosine kinase inhibitors (February 2002) were calculated up to 10 years. Hazard ratios were calculated using Cox regression models adjusted for sex, age at diagnosis and residency. An interrupted time series analysis was also performed.

**Results:**

Between 1996 and 2012, 357 new cases of chronic myeloid leukemia were diagnosed (standardized incidence rate of 1.2 per 100,000 residents), quite constant throughout the period. The interrupted time series analysis showed a gain of 40.4% in 5 years of disease-specific survival for chronic myeloid leukemia (from 47.3, 95%CI 38.5–55.5% to 80.8%, 95%CI 74.5–85.8%) after the introduction of tyrosine kinase inhibitors. The hazard ratio was 0.36 (95%CI 0.25–0.52) for cases diagnosed after tyrosine kinase inhibitor introduction, with differences per age at diagnosis: <65yo 0.17 (95%CI 0.08–0.39), >74yo 0.41 (95%CI 0.23–0.73). An improvement in survival (hazard ratio 0.66, 95%CI 0.36–1.20) was also observed in cases diagnosed before, and alive at, tyrosine kinase inhibitors introduction.

**Conclusions:**

Tyrosine kinase inhibitors increased disease-specific survival both for new and prevalent chronic myeloid leukemia cases. The effectiveness was similar to that observed in trials only in patients ages 65 years or younger.

**Electronic supplementary material:**

The online version of this article (10.1186/s12885-018-4984-3) contains supplementary material, which is available to authorized users.

## Background

Chronic myeloid leukemia (CML) is a myeloproliferative clonal disorder resulting from a neoplastic transformation of hematopoietic stem cells [1]. CML affects slightly more males than females and mainly between the fifth and sixth decades of life [2]. In Italy, the crude incidence rate is 1.61 per 100,000/year [3], and 5-year relative survival is 74% [4]. In Europe, the incidence rate is 1.10/100,000 [5].

Nowadays, the diagnosis of CML requires the detection of the BCR/ABL oncoprotein [6]. In cases diagnosed before introducing the detection of BCR/ABL as diagnostic criterion, the protein was present in 95% of CML patients [7]. The BCR/ABL oncoprotein is selectively inhibited by imatinib mesylate [8], the first tyrosine kinase inhibitor (TKI). Imatinib mesylate has transformed CML from a fatal to a chronic disease. Thanks to the results of IRIS (International Randomized Study of Interferon and STI571, [9]), imatinib was granted marketing authorization within the European Union by EMA (European Medicines Agency) in November 2001 [10] and in the USA by the Food and Drug Administration in May 2001.

According to the recommendation by the European Society For Medical Oncology (ESMO) published in 2005, imatinib became the first-line standard treatment for newly diagnosed patients with CML, with cytogenetic and molecular response to be monitored every six months [11].

However, tolerance and resistance to imatinib may develop over time [12, 13]; the failure rate at 60 months for patients receiving imatinib in the IRIS study was 17% [14].

In cases of failure of imatinib, second-line treatment is based on second-generation TKIs such as dasatinib and nilotinib [15, 16], authorized in November 2006 and November 2007, respectively [17, 18]. These new drugs were initially approved for second-line treatment through phase II studies [19], then, based on the results of phase III studies, for first-line treatment as an alternative to imatinib [20–22].

The IRIS trial showed a 5-year overall survival (OS) of 89% from the beginning of treatment [23]. A systematic review of 29 clinical trials [24] confirmed these results: an increase in 5-year survival, from 30 to 40% in the pre-imatinib period (1980–87) to 96% after the introduction of the drug (2004–2005).

European and American population-based studies showed that 5-year relative survival increased from 20 to 30% (1989–2001) in the pre-imatinib period to 50–90% (2001–2013) in the post-imatinib period [25–31]. This improvement occurred in all age groups, although older patients had a worse survival rate.

Therefore, the extent of the overall improvement varies among studies; some showed an increase in survival, such as the RCTs, while others did not obtain very satisfactory results. These differences can be explained by different drug access among patients of different ages or from different countries or by different characteristics among patients enrolled in the trials and patients in observational studies, who are a more heterogeneous group. Moreover, in a “real-world” context, delayed treatment or non-adherence to the TKI therapy is frequent and associated with critical outcomes [32–34]. Up-to-date population-based data on CML are limited [26].

The primary objective of our study is to assess the change in disease-specific survival for CML after the introduction of imatinib in first-line treatment.

The second objective is to assess potential demographic and geographic determinants of the change in disease-specific survival for CML after the introduction of imatinib.

## Methods

### Study design

This is a population-based study based on data from the cancer registries of the Italian provinces of Modena and Reggio Emilia.

### Data sources

At-risk populations, used as a denominator for trend analysis of incidence, were the resident populations in the two provinces, as recorded by the population registry of Emilia-Romagna Region on December 31 of each year considered (Source: Emilia Romagna Population by age and sex, at http://statistica.regione.emilia-romagna.it/servizi-online/statistica-self-service-1/popolazione/popolazione-per-eta-e-sesso).

Cancer cases were provided by the Modena and the Reggio Emilia provinces’ cancer registries, which routinely collect data on incident cases of cancer among all residents through the regional mortality, anatomical pathology, laboratory and hospital discharge databases. The Modena cancer registry was started in 1988, while the Reggio Emilia registry was started in 1996. The two cancer registries are validated according to national and international standards by the Italian Network of Cancer Registries (AIRTUM), the International Agency for Research on Cancer (IARC) and the International Association of Cancer Registries (IACR) and undergo annual quality and completeness control processes.

Vital status during the follow-up period and the causes of death were obtained through record linkage with Local Health Authority and mortality registries.

Data on the introduction of first- and second-generation TKIs (i.e. imatinib, dasatinib and nilotinib) in the two provinces were collected using the regional hospital pharmacy database, available since 2002.

Furthermore, individual data on drugs, delivered by hospital pharmacies to every patient, have been available since 2007. As the data are anonymous, it was not possible to link drugs to CML cases, but only to quantify the number of patients treated and to assess whether second generation TKIs were used as first- or second-line treatment. Moreover, as the use of TKIs was approved in 2005 for a number of new indications, the number of TKI users could be different from the number of CML cases.

### Setting and population

Italy has universal health coverage provided by the National Health Service through the Local Health Authorities in the 20 Regions. The provinces of Modena and Reggio Emilia are in the Emilia-Romagna Region, in northern Italy. The overall population in the two provinces on December 31, 2012 was 1,242,286 residents; 21% of the overall resident population was over age 65 years.

Healthcare in these two provinces is provided by two Local Health Authorities (with 11 hospitals), one university hospital and one cancer research hospital. The provincial hematology units are located at the university hospital in Modena (Azienda Ospedaliero-Universitaria Policlinico di Modena) and at the cancer research hospital in Reggio Emilia (Azienda USL-IRCCS of Reggio Emilia).

### Case definition

Our study included all cases of chronic myeloid leukemia (ICD-10 code C92.1 or Morphology ICDO-3 codes 98,633, 98,753, 98,763) diagnosed from 1996 to 2012 and recorded in the Modena and the Reggio Emilia cancer registries [35].

### Exposure definition

The hospital pharmacies of the two hematology units centers made their first purchase of imatinib in February 2002, three months after receiving marketing authorization from the European Medicines Agency (EMA).

Since 2002, imatinib has been authorized to treat patients affected by CML resistant to interferon-based therapy and not eligible for bone marrow transplantation [36]. Due to its high efficacy in inducing hematologic and cytogenetic remission and its better tolerance compared to interferon, imatinib has been recommended as first-line treatment since 2005 [11]. Despite this, according to the results from the IRIS RCT published in 2003 [9], the use of TKI as first-line treatment in real practice started prior to 2005, as reported for Sweden [37] and Italy.

For incident cases we considered those diagnosed after February 1, 2002, the starting date of the imatinib period. As a 6-month follow up was recommended for CML patients, we adopted the date of August 1, 2002 as the start date for changing therapy for prevalent cases and thus exposed to imatinib.

Consequently, we classified the CML cases using the date of diagnosis and vital status on August 1, 2002, according to the following scheme:

- Pre-TKI cases referred to patients who were diagnosed before February 1, 2002 and who died before August 1, 2002, and were never exposed to the new therapy;
- Prevalent cases referred to patients who were diagnosed before February 1, 2002 and who were still alive on August 1, 2002, for whom the exposure was considered only for the period after August 1, 2002, using a time-dependent variable (see statistical analysis section);
- Incident cases referred to patients who were diagnosed after February 1, 2002 and who were considered fully exposed to imatinib use.

As independent variables, we considered age at diagnosis, gender and residency (mountainous or non-mountainous area according to the Emilia-Romagna classification reported in D.G.R. no. 1734/2004 and D.G.R. no. 1813/2009), to assess whether potential geographic differences occurred in accessing the new therapy.

### Outcome and follow up

In the study period (i.e. 1996–2015), the overall mortality rate in Italy significantly decreased and the effect of TKIs on overall survival may be overestimated due to the reduction of other causes of death. Thus, we used the disease-specific survival rate as the outcome, considering both CML and potentially CML-related causes of death (i.e. other malignant or uncertain behavior, hematological neoplasms, opportunistic infections and anemia) (Additional file 1).

Vital status on December 31, 2015 was obtained through record linkage with Local Health Authority and mortality registries, which also led to the cause of death.

### Statistical analysis

First, we performed a descriptive analysis of CLM cases by sex, residency, age at diagnosis and exposure classification (i.e. Incident, prevalent or pre-TKI). The disease-specific survival rates at 1, 3, 5 and 10 years (when possible) were also calculated. Kaplan-Meier survival curves for cases diagnosed before and after imatinib introduction are also presented.

Second, to assess whether the increase in survival was due to early diagnosis, the incidence trend of CML was calculated using the join-point regression analysis and estimating the relative annual percentage change (APC) [38]. In addition, a linear regression model was used to evaluate whether there were any differences in age at diagnosis during the study period.

Subsequently, the introduction and use of TKIs in the provinces of Modena and Reggio Emilia was assessed using hospital pharmacy databases in each year of the study period. As individual data were available only from 2007, TKIs users for the preceding years were estimated applying a factor of 1/220 to overall consumption (except for the first year), which provides very similar estimates to actual users from 2007 onwards. The factor 1/220 was the actual number of doses prescribed per year per patient, as estimated by the local hospital pharmaceutical services (i.e. number of TKI users = number of TKI doses prescribed in a year / n° of doses prescribed for each patient in one year). For the first year of drug introduction (2002). the purchase of imatinib started in February for incident cases and most likely in August for prevalent cases. Therefore, a factor of 1/150, calculated proportionally to the time of exposure starting from the value of 220 per year, was used to estimate TKI users.

For our primary objective, the impact of the introduction of the drug on disease-specific survival was evaluated through interrupted time series (ITS) analysis. This method makes it possible to detect whether a specific intervention (TKI introduction) had significantly greater effect than any underlying secular trend, interrupting the previous trend and producing a level change in the outcome, i.e. disease-specific survival [39]. A sensitivity analysis of ITS using the relative survival was also performed.

A Cox model adjusted for sex, age at diagnosis and residency was used to calculate the hazard ratio for incident cases compared to cases diagnosed before the introduction of imatinib (i.e. prevalent and pre-TKI).

To evaluate the impact of the drug introduction on prevalent cases, a Cox model with time-dependent variable was used, including only cases diagnosed before TKI introduction and surviving at least a year after diagnosis. In this model, the survival time of prevalent cases was split into the period when they were considered not exposed to TKI (from diagnosis to July 31, 2002) and the period when they were considered exposed to TKI (from August 1, 2002 onwards). The length of the split survival times was limited to 5 years because there were no more exposed survival times after 2002.

The Cox models for prevalent cases were also adjusted for sex, age at diagnosis and residency.

The interactions between each independent variable and exposure variable were also assessed to explore whether the improvement in survival was different in some categories. In any case, Cox models were stratified by age class in order to evaluate whether age affected the TKI impact.

Statistical analysis was performed using Stata software, 12.0 version. ITS analysis was performed using “ITSA: Stata module” developed by Linden, Ariel [40].

### Ethics

Data were treated according to the Italian Data Protection legislation (D.Lgs.n.196/2003). The Reggio Emilia cancer registry received approval from the Provincial Ethical Committee on July 23, 2014.

This is a retrospective observational study based on cancer registry data. In accordance with the Italian privacy law, no patient or parental consent is required for large retrospective population-based studies approved by the competent Ethics Committee if data are published only in aggregated form.

## Results

**Fig. 1:**
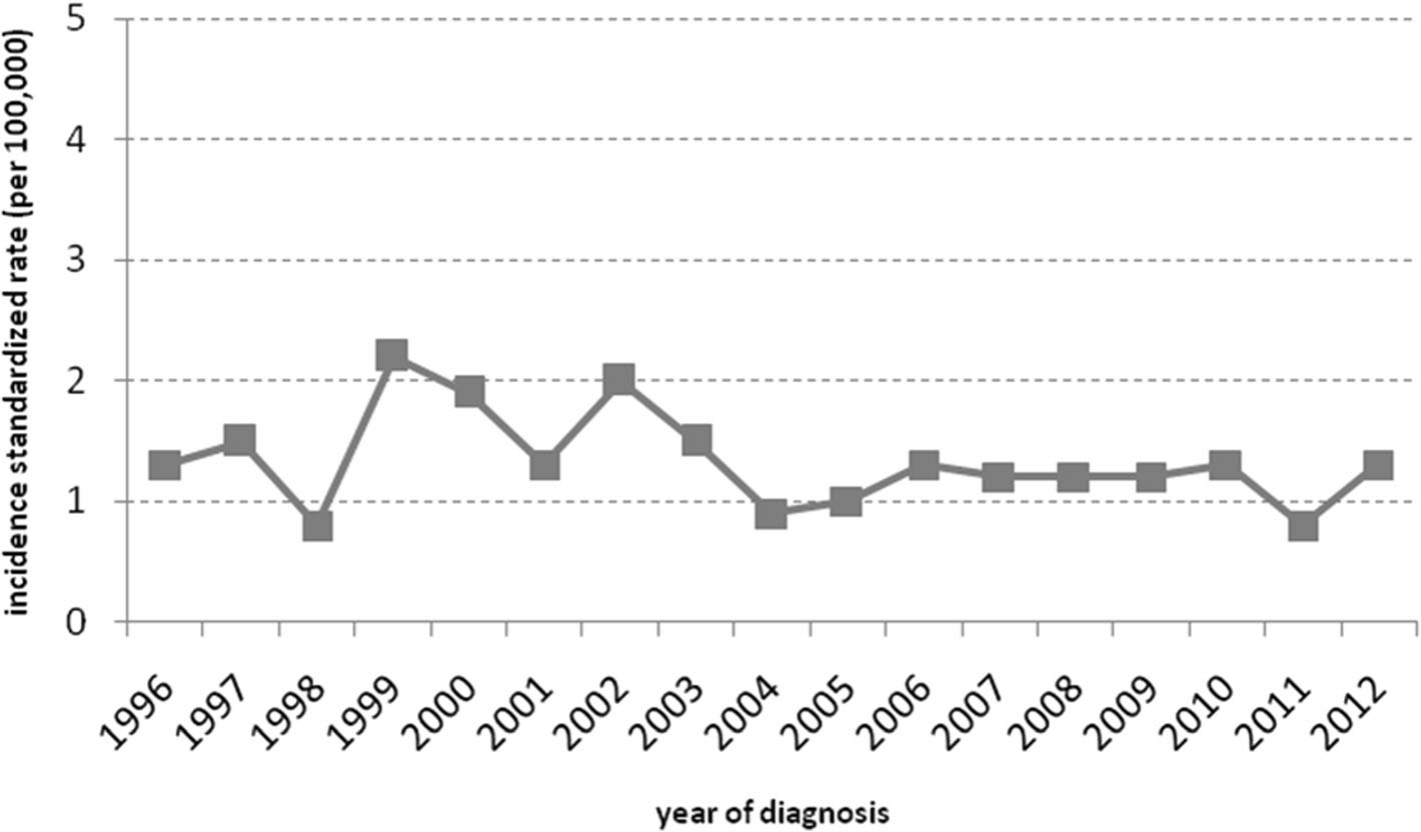
Trend in incidence rates of chronic myeloid leukemia from 1996 to 2012 in the provinces of Modena and Reggio Emilia, Italy

From 1996 to 2012, 357 new cases of chronic myeloid leukemia were diagnosed in the provinces of Modena and Reggio Emilia. The overall crude incidence rate was 1.7 for every 100,000 residents and the standardized incidence rate was 1.2 for every 100,000 residents. In the same period, the trend in incidence rates was substantially constant (APC -2.3, 95%CI -5.1-0.6) (Fig. 1) while the mean age at diagnosis was higher for cases diagnosed before drug introduction (63.4 years, 95%CI 60.3–66.5) compared to those diagnosed after February 1, 2002 (59.5 years, 95%CI 57.0–61.9)(coef = − 3.9, *p* = 0.048).

### TKI volume

**Fig. 2:**
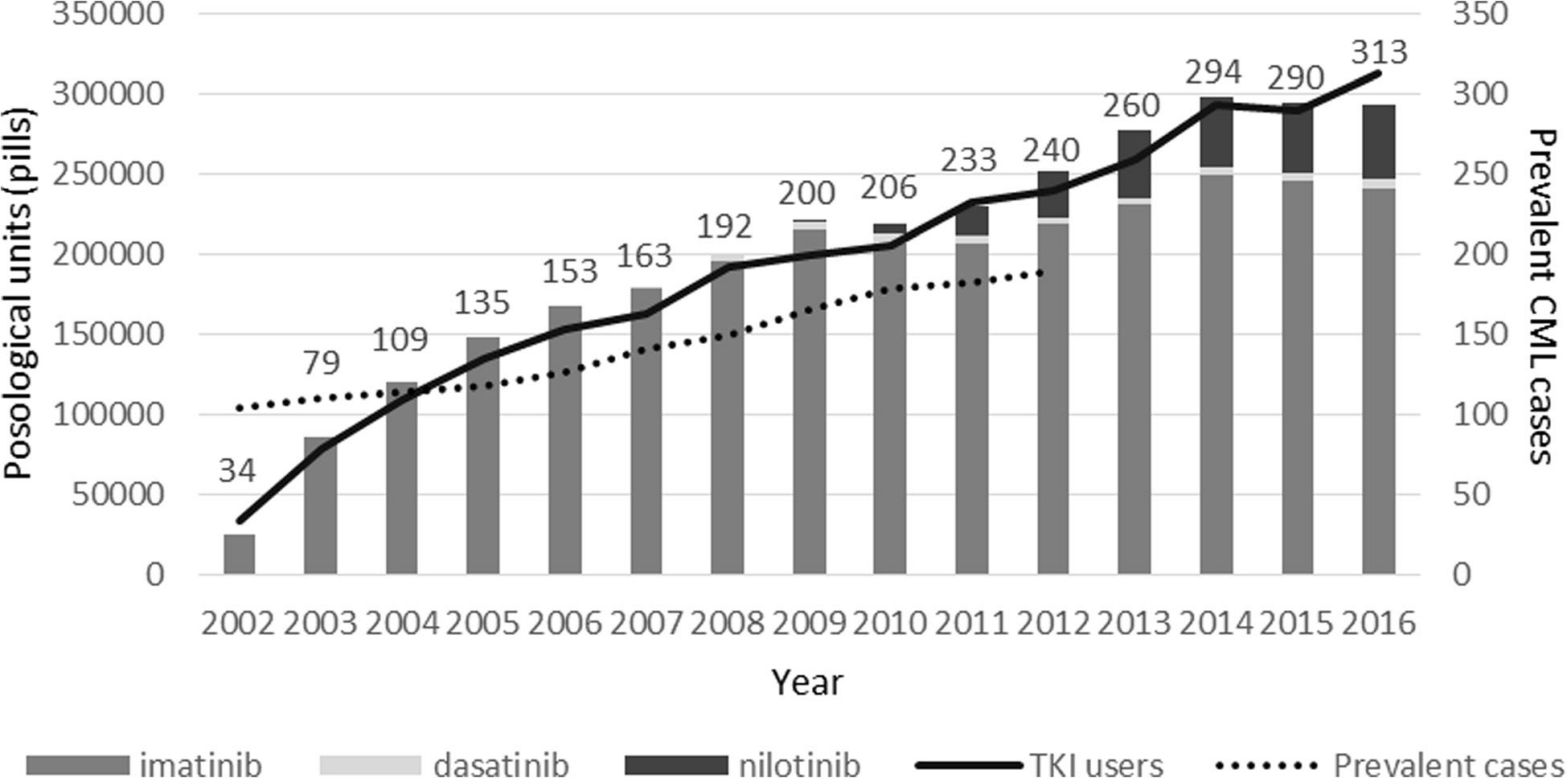
Number of prevalent cases of CML, number of TKI users (numbers on graphic area) and number of posological units (pills) of imatinib, dasatinib and nilotinib delivered per year by the hospital pharmacy to inpatients resident in the provinces of Modena and Reggio Emilia. Data from hospital pharmacy databases

Figure 2 shows the volume of TKIs administered since 2002, year of introduction of imatinib as a first-line treatment for CML, extracted from the hospital pharmacy databases of the provinces of Modena and Reggio Emilia. The use of second-generation TKIs started in 2007 for dasatinib and in 2009 for nilotinib (Fig. 2).

Moreover, the analysis of individual hospital pharmacy utilization from 2007 to 2016 showed that second-generation TKIs was prescribed as a second-line treatment in 98.3% (*N* = 116/118) of cases, in accordance with the current guidelines. The estimated number of TKI users has at least equaled the number of prevalent cases only since 2004, with a gap in 2002 and 2003.

### CML cases description and survival analysis

The characteristics of the cases are summarized in Table 1 according to the exposure variable: 75 cases were classified as pre-TKI, 73 as prevalent and 209 as incident.

**Table 1:** Chronic myeloid leukemia case description and survival analysis

CML Case Characteristics	DIAGNOSIS PRE-TKI (before February 1, 2002)				DIAGNOSIS Post-TKI (after February 1, 2002)	
	dead on August 1, 2002 (Pre-TKI cases)		alive on August 1, 2002 (Prevalent cases)		(Incident cases)	
SEX	N	%	N	%	N	%
M	48	64.0	42	57.5	117	56.0
F	27	36.0	31	42.4	92	44.0
AGE AT DIAGNOSIS						
< 65 yrs	17	22.7	40	54.8	117	56.0
65–74 yrs	23	30.7	22	30.1	46	22.0
> 74 yrs	35	46.7	11	15.1	46	22.0
RESIDENCY						
non-mountainous area	64	85.3	65	89.0	193	92.3
mountainous area	11	14.7	8	11.0	16	7.7
TOTAL	75	100	73	100	209	100
SURVIVAL	Diagnosis before February 1, 2002				Diagnosis after February 1, 2002	
	Relative Survival % (95% CI)		Disease-specific Survival % (95% CI)		Relative Survival % (95% CI)	Disease-specific Survival % (95% CI)
1 year	81.43% (73.55–87.47)		83.36% (76.21–88.53)		90.54% (85.31–94.22)	91.28% (86.52–94.42)
3 years	56.55% (47.34–65.06)		60.38% (51.69–68.00)		80.95% (74.26–86.40)	84.52% (78.70–88.86)
5 years	40.82% (31.96–49.75)		47.26% (38.49–55.53)		77.01% (69.65–83.25)	80.85% (74.49–85.78)
10 years	34.50% (25.76–43.76)		38.41% (29.90–46.85)		70.85% (61.55–79.08)	76.97% (69.52–82.82)
5-yr SURVIVAL by AGE AT DIAGNOSIS	Diagnosis before February 1, 2002				Diagnosis after February 1, 2002	
	Relative Survival % (95% CI)		Disease-specific Survival % (95% CI)		Relative Survival % (95% CI)	Disease-specific Survival % (95% CI)
< 65 yrs	66.34% (51.84–77.60)		69.08% (55.03–79.52)		92.49% (85.31–96.59)	94.63% (88.41–97.55)
65–74 yrs	39.31% (23.83–55.31)		46.77% (30.83–61.22)		67.59% (50.58–81.06)	69.01% (52.50–80.78)
> 74 yrs	3.49% (0.28–15.97)		8.05% (0.81–26.72)		40.27% (22.01–60.78)	51.21% (32.44–67.17)

Characteristics of cases of chronic myeloid leukemia diagnosed from 1996 to 2012 in the provinces of Modena and Reggio Emilia, Italy, classified by date of diagnosis (before or after February 1, 2002) and status (dead or alive) on August 1, 2002. February 1, 2002 was the date of the first purchase of imatinib by the Local Health Authorities of Modena and Reggio Emilia and August 1, 2002 was the date of the plausible full implementation of the new therapy for all the prevalent cases, according to current guidelines. Disease-specific survival was assessed for all cases with follow up to December 31, 2015. Five-year disease-specific survival stratified by age class at diagnosis was also calculated

The disease-specific survival rate for CML was higher for incident cases than for cases diagnosed before February 1, 2002, both at 3, 5 and 10 years (Table 1).

**Fig. 3:**
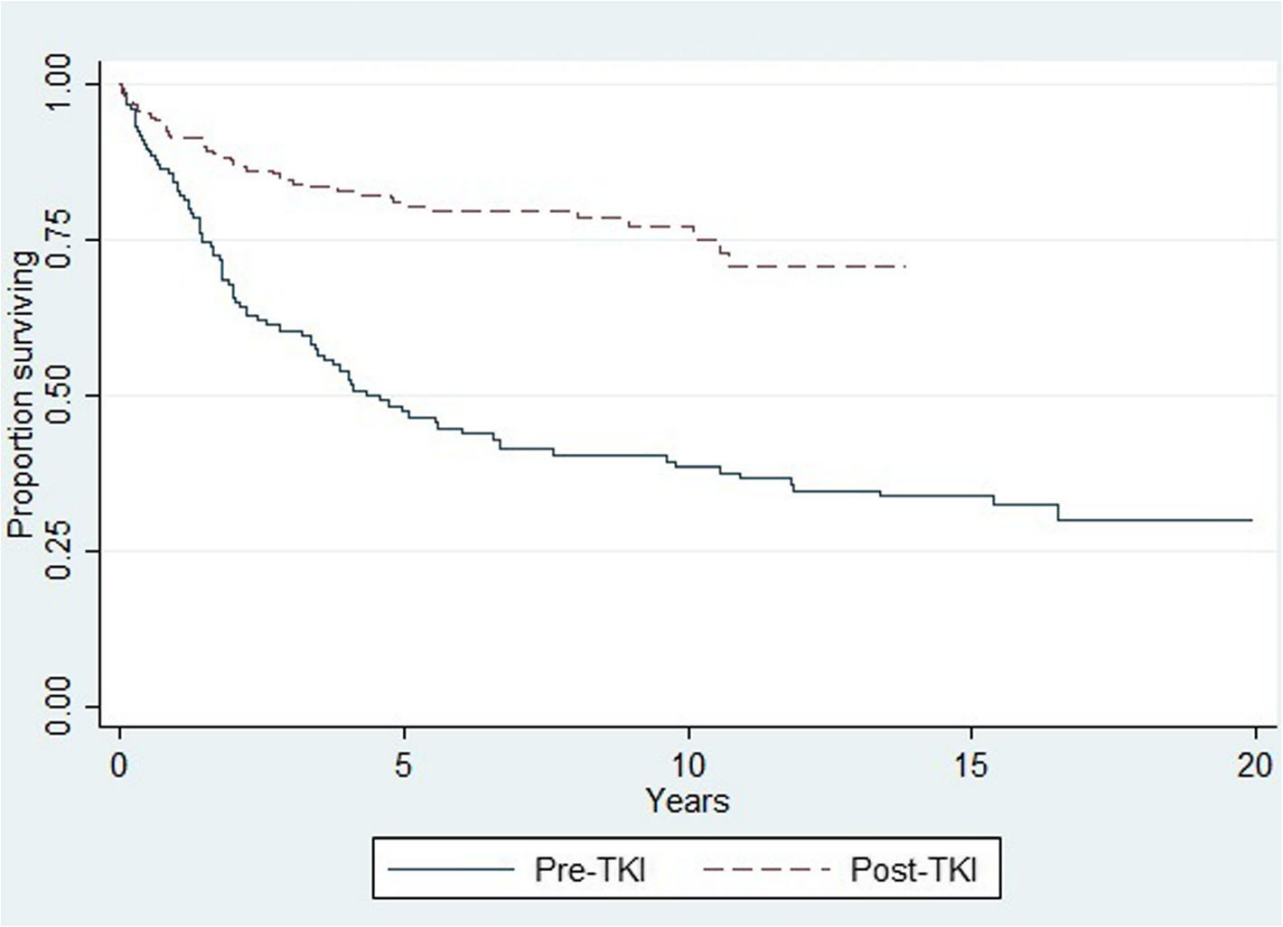
Kaplan-Meier disease-specific survival curves for chronic myeloid leukemia: Kaplan-Meier disease-specific survival curves of chronic myeloid leukemia cases diagnosed before (pre-TKI) and after (post-TKI) the introduction of imatinib on February 1, 2002

Kaplan–Meier analysis showed a difference in survival rate between cases diagnosed before and after imatinib introduction, starting from the first year and with a constant difference of almost 35% by the 10th year (Fig. 3).

**Fig. 4:**
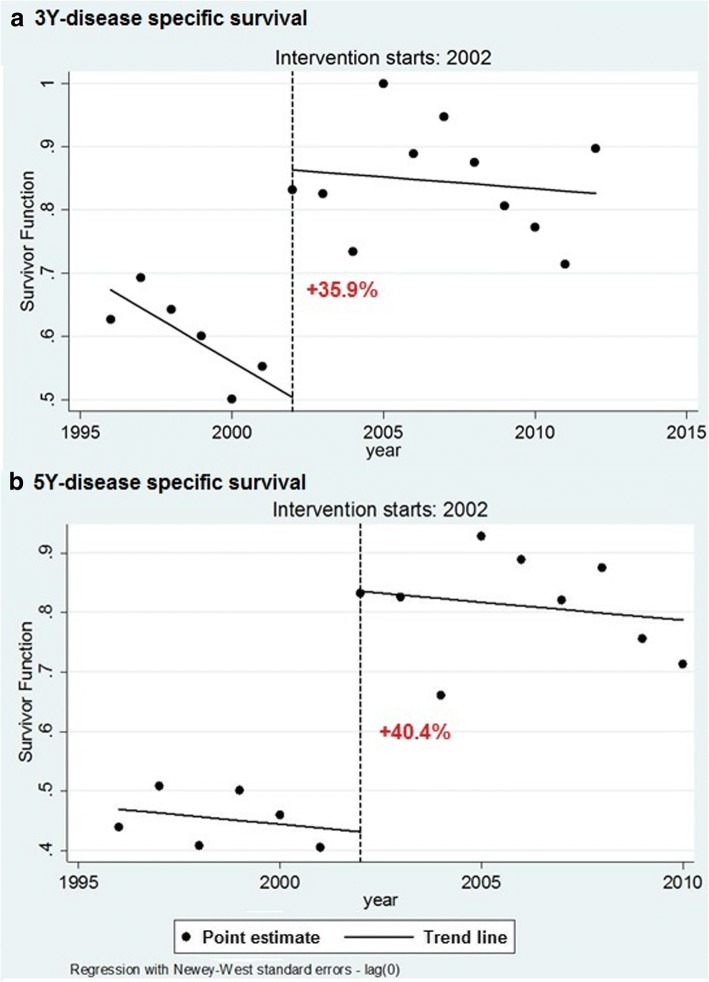
Interrupted time series analysis: The level change in 3-year (**a**) and 5-year (**b**) disease-specific (DS) survival for CML after the introduction of TKI (intervention) was calculated using interrupted time series regression models. Points represent disease-specific survival by year of diagnosis, continuous lines represent pre- and post-intervention trends, the dashed line represents the time of intervention, i.e. the drug’s introduction into clinical practice in February 2002

The ITS analysis showed a positive level change following the drug introduction of 35.9% (*p* = 0.000) on 3-year disease-specific survival (Fig. 4a), which is equal to 40.4% (p = 0.000) on 5-year disease-specific survival (Fig. 4b). After 2002, no change in trends was observed (*p* = 0.66 for 3 years; *p* = 0.53 for 5 years). The results were consistent with those of the sensitivity analysis using relative survival (data not shown).

The hazard ratios (HR), adjusted for sex, age at diagnosis and residency, showed a significant effect of the drug introduction on incident cases (HR = 0.36, 95%CI 0.25–0.52) compared to other cases.

Moreover, survival was higher for female and younger patients, while no differences were detected for residency (Table 2).

**Table 2:** Hazard ratios (HR) for incident cases

	HR	[95% CI]	
Cox model for incident cases			
Period of diagnosis			
Post-TKI vs. Pre-TKI	0.36	0.25	0.52
Sex			
F vs. M	0.65	0.45	0.93
Age at diagnosis			
65–74 yrs vs. < 65 yrs	3.46	2.19	5.47
> 74 yrs vs. < 65 yrs	8.27	5.16	13.26
Residency			
Mountainous vs. non-mountainous	1.13	0.67	1.92
Cox model for incident cases stratified by age at diagnosis			
Age at diagnosis < 65 years			
incident cases vs. non-incident cases	0.17	0.08	0.39
Age at diagnosis 65–74 years			
incident cases vs. non-incident cases	0.52	0.27	0.99
Age at diagnosis > 74 years			
incident cases vs. non-incident cases	0.41	0.23	0.73

Hazard ratios for incident cases (diagnosed after February 1, 2002) compared to other cases (diagnosed before February 1, 2002), calculated using Cox regression models adjusted by sex, age at diagnosis and residency (mountainous or non-mountainous area). Interaction between each covariate and time of diagnosis (i.e. incident or non-incident cases) was also assessed. A Cox regression model stratified by age at diagnosis and adjusted for sex and residency for incident cases was also performed

No interaction was found between the incidence period and sex, residency and age (test for interaction *p* = 0.655, 0.314, 0.161, respectively). Stratifying the model by age class at diagnosis, the effect of TKI introduction on survival was stronger in younger patients (HR = 0.17 95%CI 0.08–0.39).

Cox model performed to assess the impact of TKI introduction on prevalent CML cases, i.e. those diagnosed before February 1, 2002, showed a slightly positive effect on the risk of dying of CML in the survival period post-TKI compared to the pre-TKI period. Again, we did not find any interaction with sex and residency (test for interaction *p* = 0.731 and 0.122, respectively), while there seemed to be one with age (test for interaction *p* = 0.053), with a stronger effect for younger patients (Table 3).

**Table 3:** Hazard ratios (HR) for prevalent cases

	HR	[95% CI]	
Cox model for CML cases diagnosed before February 1, 2002			
Survived period			
Post-TKI vs. Pre-TKI	0.66	0.36	1.20
Sex			
F vs. M	0.53	0.30	0.94
Age at diagnosis			
65–74 yrs vs. < 65 yrs	1.63	0.90	2.96
> 74 yrs vs. < 65 yrs	6.14	3.12	12.08
Residency			
Mountainous vs. non-mountainous	1.20	0.55	2.59
Cox model for cases diagnosed before February 1, 2002 stratified by age at diagnosis			
Age at diagnosis < 65 years			
Post-TKI vs. Pre-TKI	0.33	0.13	0.87
Age at diagnosis 65–74 years			
Post-TKI vs. Pre-TKI	0.68	0.23	2.02
Age at diagnosis > 74 years			
Post-TKI vs. Pre-TKI	0.80	0.23	2.75

Cases diagnosed before TKI introduction. Effect of TKI introduction on prevalent cases. Hazard ratios for survival period pre-TKI and post-TKI introduction, calculated using Cox regression models adjusted for sex, age at diagnosis, residency (mountainous or non-mountainous area). Interactions between each covariate and exposure variable were assessed. A Cox regression model stratified by age at diagnosis and adjusted for sex and for residency was also performed

## Discussion

In our study, disease-specific survival for CML increased sharply after the introduction of imatinib (February 1, 2002), by 35.9% at 3 years and 40.4% at 5 years, reaching 80.9% in the post-TKI period.

The diffusion of TKI administration was quite rapid, plateauing in 2009. The estimated number of TKI users was lower than the number of prevalent cases of CML for the first two years after imatinib introduction. Moreover, since 2005, the number of TKI users was higher than prevalent cases due to the increasing number of indications of these drugs (i.e. malignant gastrointestinal stromal tumors, dermatofibrosarcoma protuberans, acute lymphoblastic leukemia, chronic eosinophilic leukemia, hypereosinophilic syndrome, myelodysplastic/myeloproliferative diseases) [41–45].

The impact of TKI use on disease-specific survival was greater on patients diagnosed after drug introduction (after February 1, 2002) but a positive effect was also seen on prevalent cases. An effect of shifting the therapy in prevalent cases has not been described before. In this population, the interaction with age is stronger with older patients diagnosed before 2002, who barely benefitted from the introduction of TKIs, probably because they never received the new treatment.

No differences were found in relation to the residency of patients, suggesting a uniform access to the new drugs in all areas, even mountainous ones, which are farther from the hematological units.

Considering age at diagnosis, our results were partially consistent with those from previous studies [25, 26], which highlighted a greater benefit for younger patients. The increase in survival was minimal in countries where TKI-treatment was not as widespread.

In a Lithuanian study, the 5-year relative survival (RS) ranged from 33% in 2000–2004 to 55% in 2005–2009, but throughout the entire study period the 5-year RS of 33% was limited to patients ages 65–74, while for those ages ≥75 it was only 18%. TKI availability (imatinib in first-line treatment) increased from 1.5% in 2000–2004 to 30.6% in 2005–2009 and was restricted to younger groups. In the same study from Lithuania, during 2000–2004, only the < 44-year age group was treated with imatinib (7% of penetrance) while in 2005–2009, the TKI penetrance of patients < 65 years was 49.5%, and until 2009, no one over age 75 received the new drug [31].

Instead, the improvement in survival was highest in previous studies from countries where first-line TKI-treatment was available to almost everyone patients. In a previous population-based Swedish study, the 5-year RS ranged from 21% (1973–79) to 80% (2001–2008) but this improvement was greater in younger patients: in the period 2001–2008, the < 50 years group showed a 5-year RS of 91% while the > 79 age group showed a 5-year RS of 25%. The use of imatinib in first-line treatment increased from 40% in 2002 to 84% in 2006 [25].

The results from another study suggest that the smaller gain in survival for older patients was not due to age-related characteristics. In fact, in the UK, the 5-year RS was 88.6% in the period between 2004 and 2013 (89.9% for patients < 60 years and 87.2% for ≥60 years) and 97% of patients received imatinib or dasatinib in first-line treatment [26]. In our study, the overall 5-year disease-specific survival was lower (80.8%), while the results were similar for younger patients (94.6% for those age < 65), with a higher gap for older patients (69.0% in patients age 65–74 and 51.2% in those age > 74). This difference could be due to a slower penetration of the new therapy in the first years after introduction, particularly among older patients.

Moreover, 5-year leukemia-related survival of 93% was observed in a population-based prospective Italian study of CML patients (> 18 years old) living in two Italian regions, diagnosed during 2008–2012 and 2010–2012, respectively [46]. In this study, 5-year disease-specific survival ranged from 94% among patients age 18–69 years to 88% for patients age 80 or older. The lower survival rate achieved by older patients in our study might be due to the different causes of death considered for estimating disease-specific survival (i.e. only deaths after documented progression to accelerated or blastic phases were considered by Castagnetti et al.). Moreover, as the Italian study period began long after the introduction of imatinib, its results were probably less affected by the slower introduction of the drug compared to our study. Future research should explore whether differences in TKI prescription in the elderly population are the cause of the lowered impact on relative survival. Finally, Castagnetti et al. reported only the results concerning cases actually treated with imatinib, as with documented Ph + CML. Due to the unavailability of the specific morphology of the older cases included in our registry, CML Ph- was also included, with a plausible reduction in the impact of the drug introduction.

### Strengths and limitations

As in any observational study with a before and after design, other factors may have changed during our study period, making the two populations (pre- and post-TKI introduction), not comparable. To support a causal link between the observed change in survival and the introduction of TKI we should rule out that other changes occurred in the epidemiology of the disease in the same time period. Early diagnosis has the potential to strongly impact survival, so we should rule out that cancers in the post-introduction period were diagnosed earlier than those in the pre-introduction period. Indeed, we observed a slight decrease in mean age at diagnosis during the study period. This change could be due to early diagnosis in the post-TKI introduction period or delay in registration in the first years of the follow-up period (i.e. the inclusion of prevalent cases, diagnosed before the start of the registry activity, in the incidence of the first years of registration, corresponding to the pre-imatinib period in our study). Nevertheless, this difference in age at diagnosis cannot explain the enormous increase in survival rate that we observed. In fact, the mean age at diagnosis decreased by 3.9 years but the distance between the two curves is much higher after the second year. Furthermore, the difference in survival curves is mostly due to a difference in the fraction of remaining patients when the curves became approximatively flat: in the post-imatinib introduction period, about 75% of patients were still alive compared to 35% in the pre-imatinib period.

The design of our study reflected the real impact on the entire population of CML patients, not considering whether they were correctly diagnosed and characterized or whether they actually received imatinib. We are measuring both the efficacy of the treatment, which is assumed in this study, and the ability of the health service to correctly treat patients with a newly available and very effective drug.

Moreover, the available routinely collected data did not make it possible to assess the real individual uptake of TKIs. Despite this, data on the delivery of TKIs in our province and the current guidelines support an almost full implementation of TKI in CML cases in the study period.

In addition, the impact of second-generation TKIs was not assessed separately, although data from hospital pharmacy databases suggest that they were only used in second-line treatment during the study period, therefore less than imatinib.

Finally, data on cytogenetics of CML were not available for the older cases included in our registry and the exclusion of the more recent CML Ph- cases might introduce a bias in the trend analysis. Therefore, we included all the CML cases in our analysis, with a potential reduction of estimated imatinib impact on survival rate. Despite this, using data from cancer registries and mortality registries allowed us to include almost all CML cases among residents in the two provinces.

## Conclusions

The spread of TKI use in Emilia-Romagna was quite rapid and caused an immediate increase in CML survival, both for new cases and prevalent ones. The impact on survival was similar in all geographic areas and in both sexes, but patients over the age of 65 years obtained a smaller gain. The 5-year disease-specific survival in patients < 65 years diagnosed in the post-TKI period reached values similar to those observed in RCTs. The effectiveness of TKIs proved to be similar to efficacy only in younger patients.

## Additional file

Additional file 1: ICD codes: ICD codes of CML and potentially CML-related causes considered as causes of death to assess disease-specific survival. (PDF 176 kb)

## Abbreviations

APC: Annual Percent Change
CML: Chronic Myeloid Leukemia
DS: Disease specific
EMA: European Medicines Agency
ESMO: European Society For Medical Oncology
HR: Hazard Ratio.
ITS: Interrupted Time Series
OS: Overall Survival
TKI: Tyrosine Kinase Inhibitor
