# Supplementary material for: The impact of introducing tyrosine kinase inhibitors on chronic myeloid leukemia survival: a population-based study
Source: BMC Cancer. 2018 Nov 6;18:1069. doi: 10.1186/s12885-018-4984-3 (PMC6219019; doi:10.1186/s12885-018-4984-3)
Supplement: Supplementary file 1 — ICD codes: ICD codes of CML and potentially CML-related causes considered as causes of death to assess disease-specific survival. (PDF 176 kb) [file 12885_2018_4984_MOESM1_ESM.pdf]

Additional file 1 – ICD Codes:

**ICD-10**	
A41	Other sepsis
C85	Other and unspecified types of non-Hodgkin lymphoma
C91	Lymphoid leukaemia
C92	Myeloid leukaemia
C97	Malignant neoplasms of independent (primary) multiple sites
**ICD-9**	
202	Other malignant neoplasms of lymphoid and histiocytic tissue
203	Multiple myeloma and immunoproliferative neoplasms
204	Lymphoid leukaemia
205	Myeloid leukaemia
208	Leukaemia of unspecified cell type
238	Neoplasm of uncertain behavior of other and unspecified sites and tissues
289	Other diseases of blood and blood-forming organs
780	General symptoms

Additional file 1 ICD codes of CML and potentially CML-related causes considered as causes of death to assess disease-specific survival.
